# *N*-acetyl-l-histidine, a Prominent Biomolecule in Brain and Eye of Poikilothermic Vertebrates

**DOI:** 10.3390/biom5020635

**Published:** 2015-04-24

**Authors:** Morris H. Baslow, David N. Guilfoyle

**Affiliations:** Nathan S. Kline Institute for Psychiatric Research, 140 Old Orangeburg Road, Orangeburg, NY 10962, USA; E-Mail: dguilfoyle@nki.rfmh.org

**Keywords:** *N*-acetylaspartate, *N*-acetylhistidine, brain, molecular water pump, magnetic resonance spectroscopy, fish, mammals

## Abstract

*N*-acetyl-l-histidine (NAH) is a prominent biomolecule in brain, retina and lens of poikilothermic vertebrates. In fish lens, NAH exhibits an unusual compartmentalized metabolism. It is synthesized from l-histidine (His) and acetyl Co-enzyme A. However, NAH cannot be catabolized by lens cells. For its hydrolysis, NAH is exported to ocular fluid where a specific acylase cleaves His which is then actively taken up by lens and re-synthesized into NAH. This energy-dependent cycling suggested a pump mechanism operating at the lens/ocular fluid interface. Additional studies led to the hypothesis that NAH functioned as a molecular water pump (MWP) to maintain a highly dehydrated lens and avoid cataract formation. In this process, each NAH molecule released to ocular fluid down its gradient carries with it 33 molecules of bound water, effectively transporting the water against a water gradient. In ocular fluid the bound water is released for removal from the eye by the action of NAH acylase. In this paper, we demonstrate for the first time the identification of NAH in fish brain using proton magnetic resonance spectroscopy (MRS) and describe recent evidence supporting the NAH MWP hypothesis. Using MRS, we also document a phylogenetic transition in brain metabolism between poikilothermic and homeothermic vertebrates.

## 1. Introduction

### 1.1. Background

*N*-acetyl-l-histidine (NAH) is a prominent biomolecule in brain, retina and lens of poikilothermic (ectothermic) vertebrates. NAH also exhibits a strong phylogenetic component in that it is a major osmolyte in the brain and eye of teleost (bony) fish, amphibians and reptiles, but is present in much lower amounts in brain and other tissues of homeothermic (endothermic) vertebrates. In birds and mammals, another acetylated amino acid, *N*-acetyl-l-aspartate (NAA) is a major osmolyte in brain and eye of these forms, and is present in lesser amounts in brain and eye of poikilothemic vertebrates. The phylogenetic distribution and metabolic relationships between NAH and NAA have been reviewed [1].

### 1.2. NAH Metabolism

NAH is synthesized from l-histidine (His) and energy-rich acetyl Co-enzyme A (AcCoA), with the acetate (Ac) derived from d-glucose (Glc) metabolism, by histidine *N*-acetyltransferase (EC 2.3.1.33) [2,3]. NAH is an unusual amino acid in that its metabolism is highly compartmentalized. In early studies of the isolated lens of the carp (*Cyprinis carpio*), it was found to be synthesized in lens and maintained at high mM concentrations, but lens cells could not hydrolyze it [4]. For its hydrolysis it was liberated to ocular fluid where NAH deacetylase (EC 3.5.1.34, now EC 3.4.13.5) [5,6,7] was present that rapidly cleaved the His which was then actively taken up by lens and re-synthesized into NAH [4]. As a result of its rapid hydrolysis, only trace amounts of NAH are present in ocular fluid *in vivo* at any given time. This cycling of NAH and His appeared to be an energy-driven pump mechanism operating at the lens/ocular fluid interface but its specific function was obscure.

### 1.3. NAH Molecular Water Pump Hypothesis

Carp lens is a typical fish lens containing about 12 mM NAH and is a highly dehydrated structure having 51.5% water and surrounded by ocular fluids having 97.9% water [4]. The content of NAH in lens of 14 fish species has been found to range from 3.3–21.7 mM [8]. In studies of goldfish (*Carassius auratus*) lens *ex vivo*, it was observed that under hypo-osmotic conditions the degree of swelling and associated cataract formation over time varied inversely with its residual content of NAH. This led to the hypothesis that the function of the NAH cycle was to remove water from the highly dehydrated lens in order to maintain its crystalline structure and clarity [9]. It was proposed that in this cycling, each molecule of NAH released from lens transported 33 obligated water molecules out of the lens into ocular fluid against a water gradient as the NAH was transported down its own gradient. In ocular fluid, both His and acetate (Ac) were released by the action of NAH acylase. His and Ac were then available for re-uptake and the liberated free water for transport out of the eye. This process wherein the facilitated export of a hydrophilic biomolecule down its gradient transports water against a water gradient across a semi-permeable membrane was termed a “molecular water pump” (MWP) and NAH was considered to be the archetype of a new class of cellular osmoregulators [9].

Simultaneously with publication of the NAH MWP paper, another paper was published in which it was demonstrated that as Glc entered cells down its gradient from extra-cellular fluid (ECF), it transported large amounts of water into cells. This process was independently called a MWP [10]. The general nature and physiological importance of both water export and import MWP’s have been reviewed [11,12,13]. In a recent study involving mammalian myocytes, it was suggested that the diffusible hydrophilic cytoplasmic buffers carnosine (Carn) or homocarnosine (Hcarn), homologues of NAH, diffusing down their cytoplasmic gradients may constitute a molecular “pump” without a membrane to actively pump Ca^2+^ against a Ca^2+^ gradient within the cytoplasm itself [14]. Thus, the principle of using the diffusion of biomolecules down their gradients to transport water and other substances against their gradients appears to be an ancient cellular innovation and an important physiological mechanism.

In this study we demonstrate that NAA and NAH can be detected separately or in mixtures of the two in phantoms as well as in brain using proton magnetic resonance spectroscopy (MRS). We also describe recent evidence supporting the NAH MWP hypothesis.

## 2. Materials and Methods

Both NAA and NAH are present in mM concentrations in the brain and lens of fish species with the content of NAH generally much higher than NAA [8]. In mammalian brain, NAA is the major osmolyte and NAH is usually present in only trace amounts [1]. These substances also present prominent MRS spectral signals due to the methyl hydrogen structures associated with their acetyl groups. Because of this, their hydrogen MRS signals are very close to one another. However, they can be distinguished from one another based on use of appropriate reference standards. NAH exhibits a strong proton spectral signal at 1.968 ppm, and NAA at 2.006 ppm (© 2011 of The Board of Regents of the University of Wisconsin System, Madison, WI, USA).

### 2.1. ^1^HMRS Methods

The data were acquired on a 7.0 Tesla Agilent (Santa Clara, CA, USA) 40 cm bore system. The gradient coil insert had an internal diameter of 12 cm with a maximum gradient strength of 600 mT/m and minimum rise time of 200 µs with customized second and third order shim coils. A Morris Instruments (Ontario, Canada) 1.5 cm inner diameter solenoid transmit coil was used for RF transmission and reception for fish brain tissue samples. A Rapid (Rimpar, Germany) volume transmit coil (72 mm ID) and both a 4 channel (rat brain) and a 2 channel (mouse brain) receive-only surface coils were used for RF transmission and reception to acquire the rodent brain spectra. The spectral acquisition consisted of a short echo time Point Resolved Spectroscopy (PRESS) sequence with the following parameters: repetition time = 4 s, echo time = 7.5 ms, number of averages = 64, number of points = 2048 and bandwidth of acquisition = 5 kHz, total acquisition time = 256 s. A 15.6 µL voxel was used for rat brain, fish tissue and phantom. A 5 µL voxel was used for the mouse brain acquisition.The number of averages was 450 for the rat spectrum and 750 averages for the mouse spectrum. The voxel used for the rat spectrum was placed in the in the prefrontal cortex. The voxel used for the mouse spectrum was placed in the in the hippocampus.

### 2.2. Phantoms

Phantoms containing NAA and NAH standards (Sigma-Aldrich, St. Louis, MO, USA) were prepared in physiological saline and placed in 5 mL sealed vials. Samples containing between 2.5 and 20 mM of each substance were prepared individually as well as in combinations of the two substances. The samples prepared were representative of the physiological range of concentrations of these substances reported to be present in brain. Typical levels of these substances found are NAH at about 10 mM in fish brain, and NAA at about 10 mM in mammalian brain.

### 2.3. Animals and Tissues

In the present study we analyzed MR spectra in brains of two fish species as well as two mammalian species. The fish species tested were the giant danio (*Danio malabaricus*) and Atlantic salmon (*Salmo salar*) and the mammals, the Sprague-Dawley laboratory rat, and the C57BL/6 laboratory mouse. Fish were obtained from commercial vendors. The giant danio brain was studied *ex vivo* and *in-situ*, the salmon brain in excised tissue, and the rat and mouse brains *in vivo* while under anesthesia. All animal procedures were performed following the National Institutes of Health guidelines with approval from the Institutional Animal Care and Use Committee at the Nathan S. Kline Institute for Psychiatric Research.

## 3. Results

### 3.1. Identification ofNAA and NAH in Phantoms Using MRS

**Figure 1:**
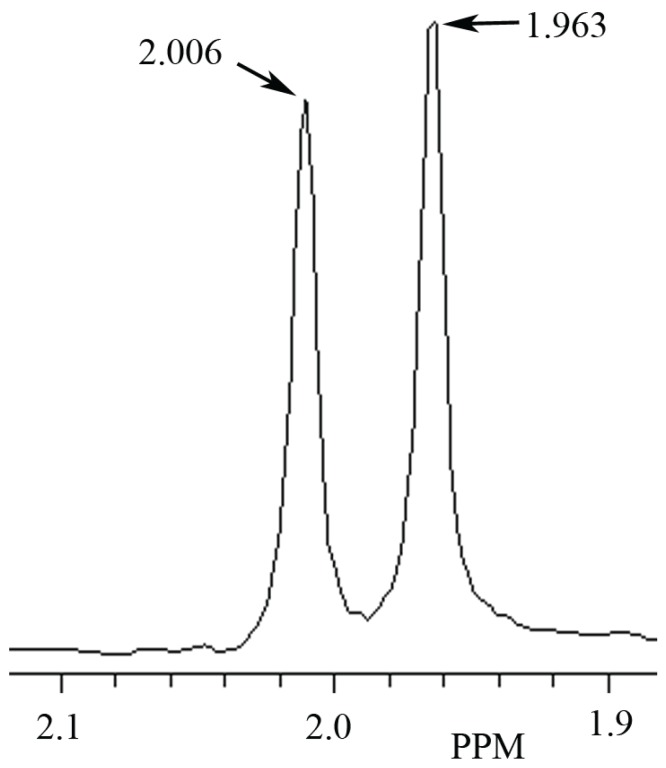
Identification of *N*-acetyl-l-aspartate (NAA) and *N*-acetyl-l-histidine (NAH) in a phantom containing 10 mM of each, and shown in the MR spectral region between 1.880 to 2.102 ppm. In this phantom the NAA peak signal is at 2.006 ppm and the NAH peak signal at 1.963 ppm.

In these studies, NAA and NAH were easily identified in phantoms containing mixtures of the two even though their assigned methyl group spectral signals are only 0.038 ppm apart. A representative proton MR spectrograph containing 10 mM each of NAA and NAH in a phantom is shown in Figure 1. MR spectral peaks in phantoms may vary somewhat from assigned values in response to differences in pH or temperature. Additional variability may be observed when measurements are made in complex mixtures such as occur in tissues or tissue extracts.

### 3.2. Identification of NAH in Fish Brain, and NAA in Mammalian Brain using MRS

**Figure 2:**
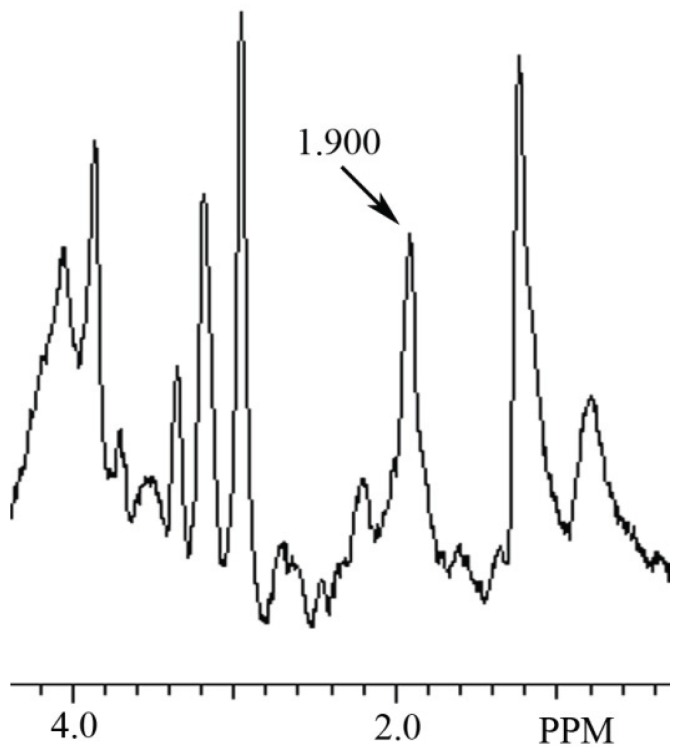
MR spectrum from giant danio brain showing a NAH peak at 1.900 ppm.

**Figure 3:**
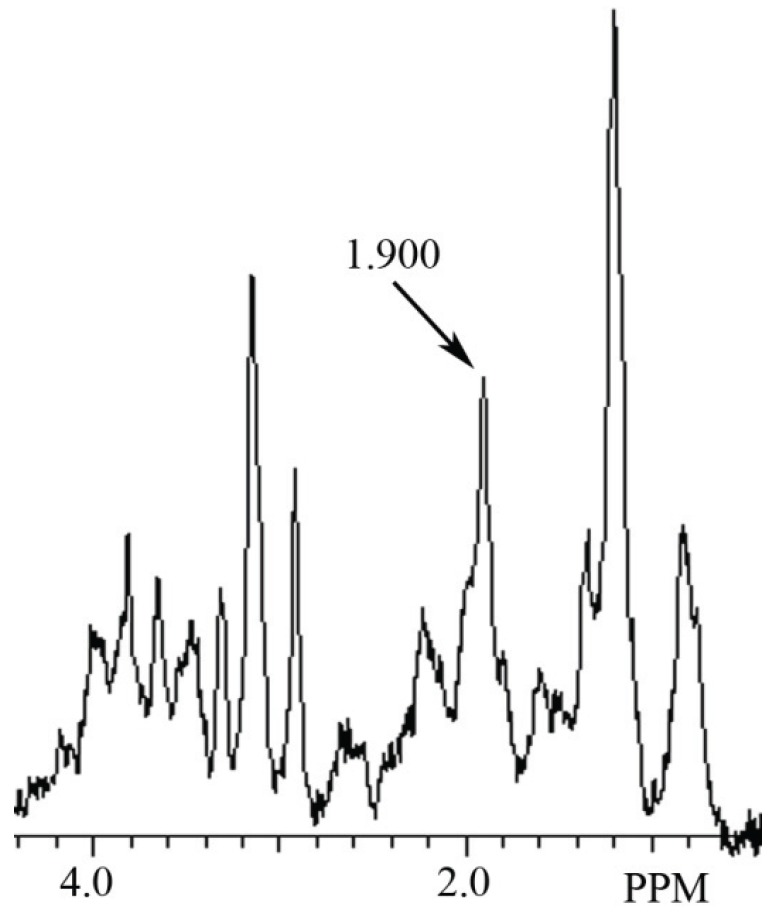
MR spectrum from Atlantic salmon brain showing a NAH peak at 1.900 ppm.

**Figure 4:**
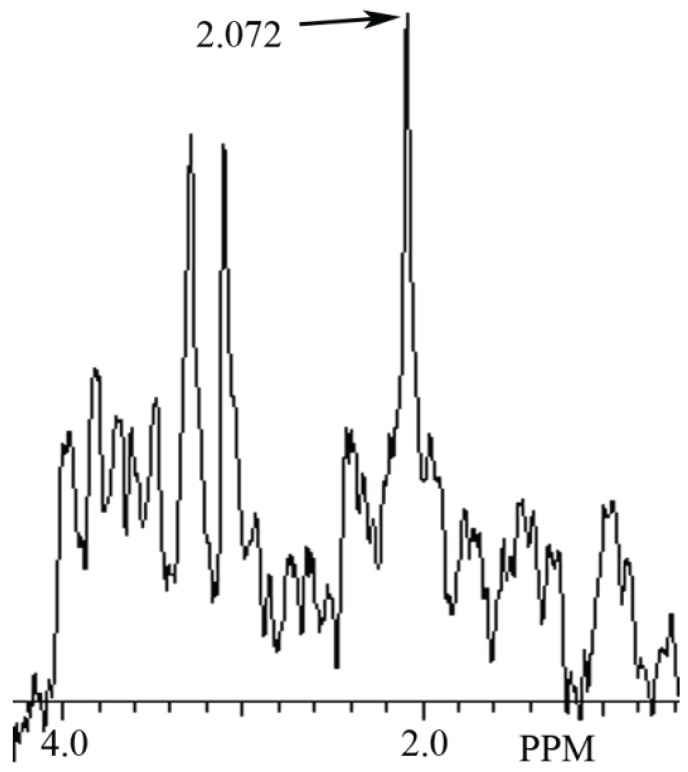
MR spectrum from rat brain prefrontal cortex showing a NAA peak at 2.072 ppm.

**Figure 5:**
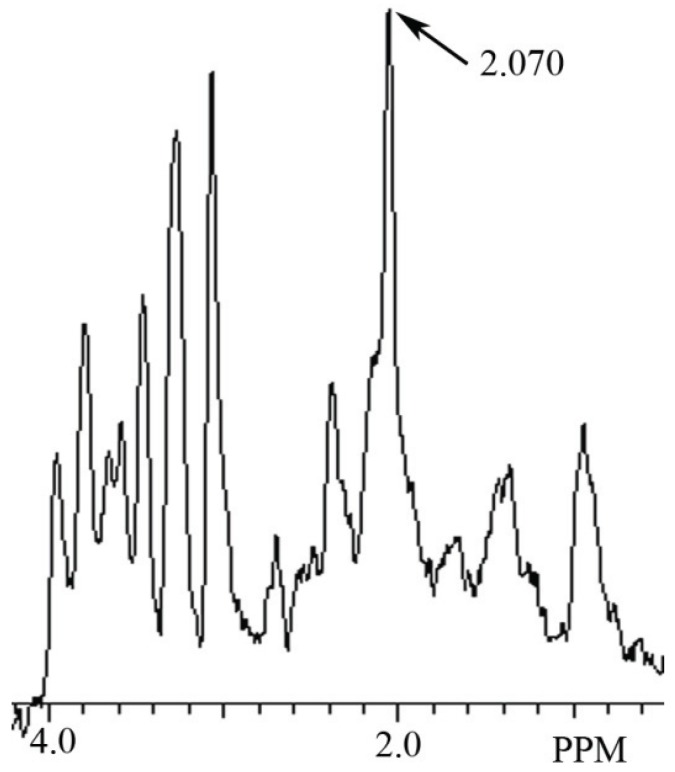
MR spectrum from mouse brain hippocampus showing a NAA peak at 2.070 ppm.

In this study a NAH MR spectral peak was clearly identifiable in fish brain, but not in mammalian brain. Conversely, a NAA MR spectral peak was clearly identifiablein mammalian brain, but not in fish brain. In Figure 2 we show the MR spectrum obtained from giant danio brain, and in Figure 3 from Atlantic salmon brain. In Figure 4 we show the MR spectrum obtained from rat brain, and in Figure 5 the MR spectrum from mouse brain.

## 4. Discussion

### 4.1. The Use of MRS to Determine the Presence of NAH in Fish Brain

In the present study, based on use of NAH and NAA standards, we identify a MRS signal peak at 1.963 ppm as NAH and a MRS signal peak at 2.006 ppm as NAA and show that NAA and NAH can be identified in mixtures of these biomolecules in phantoms. Further, we demonstrate for the first time using proton MRS, the detection and identification of NAH in the brains of fish and its paucity in the brains of mammals. We hope that these findings will prove to be useful in further studies of the hypothesized physiological MWP functions of these enigmatic amino acids.

In a MRS study of the brain of the zebra danio (*Brachydanio rerio*) *in vivo* and in its brain extracts [15], it was reported that only NAA was detected. Of interest, the MRS signal identified as NAA in the living zebra danio specimen appeared to be at about 1.97 ppm, downstream of the NAA signal, and the assigned MR spectral signal of NAH. Since in the zebra danio paper there was no citation regarding the presence of large amounts of NAH in fish brain, its absence was apparently overlooked. Furthermore, the automated LC model program [16] used to identify and quantify the spectral peak appearing at about 1.97 ppm in the paper was developed for mammalian brain which contains only trace amounts of NAH. Thus, there is good reason to believe that the NAH peak in this *in vivo* study [15] was misidentified by the automated program as NAA. In this same study, an extract of the zebra danio brain was also analyzed using high resolution MRS. From the published spectrograph showing much better resolution, it is even more evident that the peak observed at about 1.97 ppm is probably NAH, which had previously been shown to be present in the zebra danio using an independent chromatographic method [17]. 

In another study, the metabolites present in goldfish brain extracts were analyzed using MRS [18]. In this study the assigned resonances were taken from a number of cited spectral reference programs. In their paper a strong signal was observed at 1.98 ppm, but based on the spectral documentation programs used, this peak was assigned to acetamide whose spectral peak is about 1.99 ppm. In this same report the assigned NAA spectral signal was clearly distinguishable at 2.02 ppm and so identified. Once again, in the literature cited in this paper there was no reference to any publication regarding the large amounts of NAH known to be present in fish brain. Previously, in similar extracts of goldfish brain, NAA and NAH were analyzed using high pressure liquid chromatography (HPLC) and were found to be present at 3.10 and 8.28 mM respectively [8] which would correspond with the MR signal peaks at 2.02 and 1.99 ppm that had been observed.

In summary, the known NAH spectral peak at about 1.97 ppm in these MRS studies of fish brain had been readily detected [15,18] but misidentified by spectral programs that apparently did not contain a NAH MR spectrum in their spectral libraries. In addition, its absence was probably overlooked because, based on their published literature citations, there was no evidence of any awareness of NAH or of its high mM content in fish brain.

### 4.2. Recent Evidence in Support of the NAH Efflux MWP Hypothesis in Fish Lens and Eye

The development of lens cataracts in Atlantic salmon under aquaculture conditions was described in 2005 as being a long-standing problem [19]. Since diets of these fish in culture are controlled, the dietary content of His was selectively increased from 11.7 g/kg of diet to 18.1 g/kg, an increase of about 65% in available His. In this experiment [19], His was the only amino acid of 16 amino acids in the diet that was increased. Based on the His supplementation protocol, there was a positive effect on reducing cataract formation in this population. In addition, the His diet-supplemented fish had double the content of His in the lens at about 2.5 mM, and lens NAH had risen to about 12 mM. Thus, the increase in lens NAH and decrease in lens cataract formation were highly correlated. While these researchers could not specifically identify their cataract findings as a function of the MWP hypothesis [9], they considered it as one of several possibilities. Similar published findings in 2009 [20] also supported a role for NAH in lens water homeostasis but concluded that the exact mechanism was unknown.

In 2010, it was observed that levels of NAH in lens of salmon are much higher in seawater fish when compared to freshwater fish and it was proposed that NAH was the major osmolyte in lens which balances increases in aqueous humor osmolality in response to changes in the seawater environment [21]. They also observed that fish fed a His-enriched diet not only had increased lens NAH levels, but that NAH efflux in *ex vivo* cultured lenses was stimulated by hypo-osmotic exposure. This observation directly supported the findings in the MWP hypothesis study [9]. However, these authors went on to conclude that NAH, the major osmolyte in fish lens, functioned as an exportable osmolyte to extra-cellular (ocular) fluids in order to balance extra-cellular osmolarity. Based on this hypothesis, they explained that there was no evidence in their *ex vivo* study regarding the role of NAH cycle to “support its role as a molecular water pump”. This conclusion is inconsistent with known NAH metabolism in the eye since NAH released to ocular fluid *in vivo* is rapidly hydrolyzed by ocular fluid NAH acylase so that only trace amounts of NAH are ever present in ocular fluid of living fish at any given moment [4]. Therefore, under normal physiological conditions NAH is not available for this proposed osmotic purpose.

In a subsequent paper [22], salmon lenses were cultured *ex vivo* for 96 h in an enriched His medium. In this medium the His level was 0.57 mM. After the incubation period the lenses in this medium had actively taken up His against a His gradient and these lenses had increased their His levels three-fold from 0.5–0.7 mM to a value of 1.7 mM. In these same lenses NAH levels also increased three-fold from 2.3–2.6 mM to 6.5–8.2 mM and moreover these lenses were observed to gradually release NAH to the support medium, which in this case had no NAH acylase to hydrolyze it. While the results of this study clearly indicated that His was taken up by the lenses against a His gradient, and that His was synthesized into NAH, which in turn was released to the culture medium as had been previously reported [4], these authors proposed that the primary function of NAH in salmon lens was that of an “antioxidant”. A recent study further supports the findings that increased His in the diet of salmon and increased lens NAH reduce the prevalence and severity of cataract formation [23].

Taken together, these studies strongly corroborate all of the empirical evidence presented in the original NAH cycling and NAH MWP hypothesis papers [4,9]. They support the finding that the level of NAH in the lens is inversely related to the incidence of cataract; that isolated lenses swell under hypo-osmotic conditions and are protected by high NAH levels; that His is actively taken up by lenses against a His gradient and synthesized into NAH, and lastly that the NAH is gradually released over time to the culture fluid. One problem with analyses of the results of the *ex vivo* studies and of some conclusions drawn by the authors is that the role of the NAH synthetic and hydrolytic enzymes and their unusual compartmentalization *in vivo* was not considered. The metabolism of NAH in the eye *in vivo* is a dynamic process and its physiological role only becomes evident when the entire cycle is present.

## 5. Conclusions

### 5.1. Identification of NAH in Fish Brain Using MRS

In this article we show for the first time using MRS that NAH, an important osmolyte in poikilotherm brain, can be readily detected and identified in the brain of bony fish. We also show that NAA, a major osmolyte in homeotherm brain, can be easily detected and identified in mammalian brain using this same technique. The phylogenetic shift in the relative content of these brain metabolites between poikilotherms and homeotherms has been known for decades [1].In this study using MRS we have now been able to clearly visualize this evolutionary change in brain metabolites (Figure 2, Figure 3, Figure 4 and Figure 5). In addition, we present brain MR spectra for two additional fish species, the Atlantic salmon and the giant danio.

### 5.2. Support for the NAH MWP Hypothesis

The identification of the NAH lens/ocular fluid cycle in the fish lens [4], and the hypothesis that NAH functioned as a MWP [9] were based on empirical evidence. In the recent studies of NAH metabolism in salmon eye, all of the experimental results in both previous studies have now been verified. While any hypothesis always remains open to question and reinterpretation, these recent fish lens studies strongly support the original empirical evidence that led to the hypothesis that NAH functioned as a MWP. In addition, other independent studies showing that MWP’s constitute an important physiological mechanism in animals also lend support to the hypothesis that the function of the NAH cycle as described in the fish eye was probably correct and that it represented the archetype efflux MWP.

### 5.3. A Problem of Metabolite Identification and Quantification when Using Automated MRS Programs in Different Species

In this article we identify a problem inherent in relying on automated MR spectral programs for identification and quantification of metabolites in tissues and their extracts. All such programs rely on spectral data associated with known standards, with substances in complex mixtures tentatively identified by their primary spectral signal position, and their quantification based on peak signal strength. Our studies of phantoms containing standards of the biomolecules NAA and NAH present in brain indicate that automated programs, which by their nature have limited MR spectral libraries for the identification of MRS peaks, cannot be assumed to work in all cases unless all reference data sets of all the metabolites present in the sample are included in the analysis. This is especially true where they are applied in studies of poikilothermic organisms including fish, amphibians, reptiles and other forms that may express different metabolic pathways and have unusual end products, some of which may have spectral peaks at or near those present in documented reference sets. In the present study we have shown that NAH, the major acetylated amino acid in fish brain can be identified in giant danio and Atlantic salmon brain using MRS even though it has a spectral signal very near that of NAA. Conversely, NAH can be misidentified using automated spectral programs specifically designed for homeothermic organisms.

### 5.4. Retrospective Identification of NAH in Published Fish Brain MRS Studies

In the present study, by analysis of phantoms containing NAA and NAH standards and based on specific knowledge of the presence of NAH in both the zebra danio [17] and goldfish [8], we are able to show that NAH, by its MRS spectral profile and relative signal strength, can be identified retrospectively with a large degree of certainty in MR spectrums of brain extracts of the zebra danio [15] and goldfish [18] in previously published reports.

### 5.5. The Thermal Dimension in Poikilotherms Provides a Unique Investigative Tool to Study the Physiological Functions of NAH and NAA

In homeotherms, the physiological function of NAA has been investigated using neurostimulation as a probative tool in order to alter its content in brain [24]. Poikilotherms must adapt to short-term diurnal and longer-term seasonal changes in ambient temperature in order to survive. In response to this environmental variable, it has been observed that in fish, concentrations of many brain metabolites change significantly during adaptation [25]. In killifish (*Fundulus heteroclitus*), after acclimation to 27 °C from an ambient of 13.3 °C for 13 days, brain NAH levels dropped by about 50% in the warm adapted fish. In goldfish acclimated to 15 and 30 °C from an ambient of 25 °C for 15 days, the warm adapted fish again had only half the level of NAH in their brain as cold adapted fish. This inverse relationship between adaptation temperature and brain NAH content in poikilotherms presents a unique opportunity to further probe the physiological roles of NAH and NAA in the vertebrate brain using a novel investigative tool not available in homeotherms.

### 5.6. A Possible Dietary Approach for Treatment of some Brain and Eye Disorders in Homeotherms

In the dietary His supplementation studies [19,20], a therapeutic role for the essential amino acid His in synthesis of NAH and preventing lens cataract formation in teleost fish was reported. This observation may have relevance to homeotherms where NAH and the His dipeptides, Carn and Hcarn are present in cultured rat neurons [26] and Carn and Hcarn are important biomolecules in human brain and eye.

## Abbreviations

Ac: Acetate
AcCoA: Acetyl Co-enzyme A
Carn: Carnosine
ECF: Extra-cellular fluid
Glc: d-glucose
His: l-histidine
Hcarn: Homocarnosine
HPLC: High pressure liquid chromatography
MWP: Molecular water pump
MRS: Magnetic resonance spectroscopy
NAA: *N*-acetyl-l-aspartate
NAH: *N*-acetyl-l-histidine
